# Tirzepatide for Recurrent Weight Gain after Bariatric Procedures: Real-World Evidence of Efficacy and Safety

**DOI:** 10.1007/s11695-026-08754-7

**Published:** 2026-06-05

**Authors:** Federica Vinciguerra, Carla Di Stefano, Fabio Guccione, Claudia Degano, Stefania Nigro, Roberto Baratta, Luigi Piazza, Giuseppe Navarra, Lucia Frittitta

**Affiliations:** 1 Department of Clinical and Experimental Medicine, University of Catania, Catania, Italy; 2 General and Emergency Surgery Department, ARNAS Garibaldi, Catania, Italy; 3 Department of Human Pathology, Azienda Ospedaliera Universitaria Policlinico “G. Martino”, Messina, Italy; 4 Endocrinology Unit, ARNAS Garibaldi, Catania, Italy; 5 Diabetes and Obesity Center, ARNAS Garibaldi, Catania, Italy

## Abstract

**Background:**

Recurrent weight gain after bariatric surgery (BS) or endoscopic bariatric therapy (EBT) remains a long-term clinical challenge, potentially undermining long-term treatment success. Tirzepatide, a dual agonist of the glucose-dependent insulinotropic polypeptide (GIP) and glucagon-like peptide-1 (GLP-1) receptors, has shown promising results in obesity treatment, but data regarding its use in post BS or EBT recurrent weight gain are limited.

**Methods:**

This observational cohort study evaluated the effectiveness and safety of tirzepatide in patients with recurrent weight gain after BS or EBT. Anthropometric data, BMI categories, and adverse events were collected at baseline and after 24 weeks of treatment.

**Results:**

A total of 34 patients (26 females, 8 males) who experienced recurrent weight gain after BS (*n* = 32) or EBT (*n* = 2) were treated with once-weekly subcutaneous tirzepatide (dosing range 2.5–10 mg/week). After 24 weeks, the mean percentage total body weight loss (%TBWL) was 18.1 ± 5.6% (*p* < 0.0001), with a significant reduction in waist circumference (*p* < 0.0001). Most patients shifted to lower BMI categories, with the majority reaching overweight or normal weight status. Adverse events were exclusively gastrointestinal (constipation, diarrhea, nausea), generally mild, and did not lead to treatment discontinuation.

**Conclusion:**

Preliminary evidence suggests that tirzepatide is associated with significant weight loss and good tolerability in this observational cohort for managing recurrent weight gain after bariatric surgery and endoscopic bariatric therapy. These findings suggest a potential role for tirzepatide as a valuable nonsurgical option in the multidisciplinary management of patients experiencing recurrent weight gain after bariatric procedures.

**Supplementary Information:**

The online version contains supplementary material available at 10.1007/s11695-026-08754-7.

## Key Points

Post-bariatric weight regain remains a significant clinical challenge.

Tirzepatide is associated with substantial weight loss in patients with recurrent weight gain after bariatric surgery and endoscopic bariatric therapy.

Tirzepatide appears to have a favorable safety profile in the post-bariatric setting.

**Supplementary Information:**

The online version contains supplementary material available at 10.1007/s11695-026-08754-7.

## Introduction

Bariatric surgery (BS) remains the most effective long-term intervention for obesity, providing the greatest weight loss and improvements in metabolic and cardiovascular-related medical problems, as well as enhancing quality of life and life expectancy [1–3]. However, obesity is a chronic and relapsing condition. Over time, some patients experience recurrent weight gain which may compromise surgical outcomes and lead to the recurrence of obesity-related complications, affecting both physical and mental health.

The amount of recurrent weight gain after BS varies depending on the definition, type of surgery, and the length of follow-up [4]. Studies report that 20–25% of patients experience significant weight gain within 2–5 years after surgery [5].

In recent years, endoscopic transoral bariatric therapies (EBT) have been established as minimally invasive modalities for the management of obesity. Nonetheless, clinical evidence indicates that approximately 25% of patients experience weight regain within the first 24 months following endoscopic sleeve gastroplasty (ESG).

Managing recurrent weight gain after BS and EBT presents unique challenges that require specialized approaches, considering altered gastrointestinal anatomy and physiology.

Current treatment options for recurrent weight gain after BS and EBT include intensive lifestyle changes, nutritional counseling, psychological support, obesity management medications, and revisional surgery [6–8]. Among medications, GLP-1 receptor agonists have shown moderate effectiveness, with liraglutide providing an additional 5–10% weight loss in post-bariatric patients [7]. Recently, tirzepatide, a once-weekly subcutaneous peptide, dual agonist targeting glucagon-like peptide-1 (GLP-1) and glucose-dependent insulinotropic polypeptide (GIP) receptors, has been approved for obesity management, due to its significant weight loss results in non-surgical groups [9]. However, limited retrospective data are available on its safety, tolerability, and effectiveness after BS [10–12]. This study aims to assess the real-world effectiveness and tolerability of tirzepatide in patients experiencing recurrent weight gain after various bariatric procedures.

**Fig. 1:**
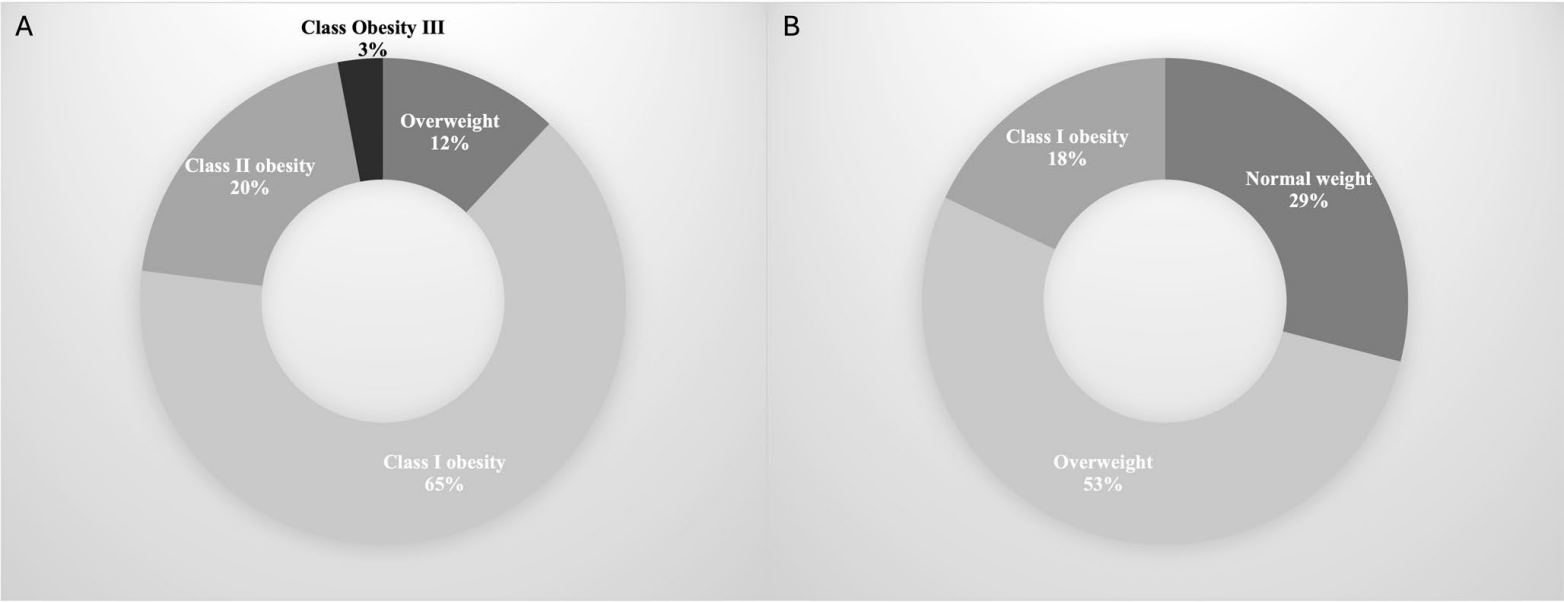
Shift in BMI category before and after treatment

## Materials and Methods

This prospective observational study was conducted from November 2024 to July 2025 at a single tertiary referral center. During the examined period, 55 patients with recurrent weight gain were referred.

Recurrent weight gain was defined as a weight gain of at least 15% of the weight lost after BS and EBT. Weight gain was calculated using the following formula: (current weight − nadir weight) / (pre-bariatric weight − nadir weight) × 100.

All were assessed for potential surgical causes of recurrent weight gain. The clinical decision to proceed with nutritional intervention, pharmacotherapy, or revisional bariatric surgery was based on a multidisciplinary assessment, including considerations of surgical risk and patient preference.

**Fig. 2:**
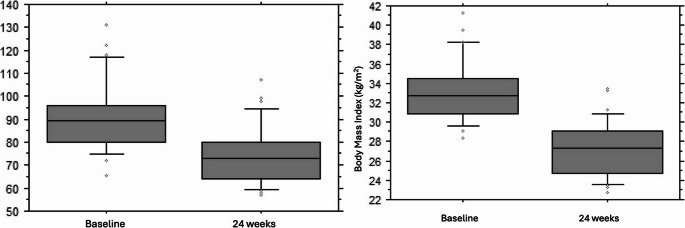
Boxplot of changes of body weight and BMI before and after treatment

In Italy, anti-obesity pharmacotherapy is not reimbursed, and economic factors were also taken into account.

Eligibility was based on clinically meaningful post-bariatric weight recurrence from the nadir. Additionally, independently of BMI, the recurrence of weight-related psychological, mechanical, or metabolic complications was considered in therapy initiation decisions.

Exclusion criteria included: use of other obesity management medications, pregnancy or lactation, severe organ dysfunction, type 1 diabetes, NYHA class IV heart failure, history of pancreatitis, or personal/family history of medullary thyroid carcinoma or MEN2 syndrome.

In addition, patients with type 2 diabetes were excluded because, at the time of data collection, tirzepatide was not reimbursed in Italy for individuals with diabetes. This criterion also reduced confounding and improved population homogeneity, as type 2 diabetes is a well-established predictor of an attenuated weight-loss response to both pharmacological and procedural obesity treatments.

The study protocol was approved by the Ethics Committee (14/CECT2, 15/10/2024) and conducted according to the Declaration of Helsinki principles.

The final sample comprised 34 patients who had undergone bariatric procedures, including surgical procedures—one-anastomosis gastric bypass (OAGB, *n* = 18, 53%), sleeve gastrectomy (SG, *n* = 13, 38%) and adjustable gastric band (AGB, *n* = 1, 3%)—and endoscopic procedures—endoscopic sleeve gastroplasty (ESG, *n* = 2, 6%).

The mean time since the bariatric procedure was 6 ± 3 years.

Tirzepatide was started at 2.5 mg and titrated in 2.5 mg increments to reach the minimum effective dose according to the drug’s summary of product characteristics. As treatment costs were out-of-pocket, a pragmatic “minimum effective dose” strategy was adopted to minimize expenses while preserving clinical effectiveness.

The maintenance doses used ranged from 2.5 to 10 mg/week: 58.8% (*n* = 20) received 5 mg/week, 32.3% (*n* = 11) 7.5 mg/week, 5.8% (*n* = 2) 10 mg/week, and 2.9% (*n* = 1) 2.5 mg/week. Dose escalation followed the approved titration scheme and was individualized based on tolerability (particularly gastrointestinal adverse events), weight-loss response, and patient preference. The mean maintenance dose achieved across participants was 6.0 ± 1.6 mg/week.

All participants received nutritional counseling from a registered dietitian and were prescribed a Mediterranean diet characterized by a high intake of plant-based foods rich in fiber, low-glycemic-index carbohydrates, and 1.5 g of protein per kilogram of ideal body weight, with extra-virgin olive oil as the primary fat source. Patients were also instructed to maintain moderate-intensity physical activity for at least 150 min weekly.

Physical examinations and anthropometric measurements (weight, height, waist circumference) were performed. A total of 5 follow-up visits occurred between baseline and 24 weeks: at weeks 4, 8, 12, 16, and 24 post-baseline, in line with standard clinical practice for GLP-1/GIP agonists to monitor titration tolerance. BMI was calculated, and patients were classified by standard BMI categories [13]. Adverse event collection and monitoring were conducted through both active solicitation and spontaneous patient-reported incidents during scheduled clinical visits.

### Statistical Analysis

Continuous variables are expressed as mean ± standard deviation (SD), while categorical variables were provided as number and percentage. Changes from baseline were analyzed using paired t-tests for continuous variables and chi-square test for categorical variables.

Exploratory subgroup analyses were performed to describe % total body weight loss (%TBWL) at week 24, stratified by bariatric/endoscopic procedure type and by the maintenance tirzepatide dose achieved at week 24. In addition, exploratory linear regression models were fitted with %TBWL at week 24 as the dependent variable to assess the association with maintenance dose (mg/week). Maintenance dose was considered the primary independent variable. The multivariable model also included age, sex, procedure type, and baseline BMI. To reduce potential multicollinearity or redundancy among anthropometric predictors, baseline body weight was not included alongside baseline BMI. Given the limited sample size and the individualized, non-randomized titration strategy, these analyses were considered hypothesis-generating.

The sample size was calculated as follows. The study was powered for the primary endpoint of percent total body weight loss (%TBWL) at 24 weeks. We considered a clinically relevant threshold of 5% TBWL and expected a mean %TBWL of 10% with a standard deviation (SD) of 6% for the paired change, based on recent post-sleeve cohorts treated with tirzepatide or semaglutide [11]. Using a two-sided α = 0.05 and 90% power for a one-sample paired mean test, the required sample size was n = ((Zα/2 + Zβ)·SD/Δ)^2 = ((1.96 + 1.28)·6/5)^2, approximately 16 participants. With a 20% attrition rate, the target enrollment was *n* = 20. The final analyzed sample (*n* = 34) exceeded this target and provides over 99% power to detect a mean %TBWL of 10% or more versus the 5% threshold. Statistical significance was set at *p* < 0.05.

## Results

### Baseline Characteristics

The study involved 34 patients, of whom 76% were female (F/M ratio: 26/8). The mean age was 49.1 ± 11.1 years. The mean body weight was 90.8 ± 14.7 kg, and the mean BMI was 33.2 ± 3.2 kg/m², with no significant differences between genders. The population’s baseline characteristics are shown in Table S1.

At baseline, 65% (*n* = 22) had Class I obesity, 20% (*n* = 7) had Class II obesity, and 3% (*n* = 1) had Class III obesity. Four patients (12%) were overweight; none had a normal BMI (Fig. 1A).

### Weight Loss and BMI Shift

After 24 weeks of treatment with tirzepatide, the percentage of total body weight loss (%TBWL) in the entire cohort was 18.1 ± 5.6%, with no significant differences by sex or surgical type.

In particular, weight loss percentages were similar between OAGB and sleeve gastrectomy (19 ± 5.4% vs. 18.5 ± 5.4%, respectively, *p* = 0.79). ESG (*n* = 2) and AGB (*n* = 1) patients appeared to have lower weight loss (11.5 ± 1.9% and 9.1%, respectively); however, these observations are only descriptive, as the very small sample sizes preclude statistical analysis.

When stratified by maintenance tirzepatide dose achieved at week 24, a numerical gradient in %TBWL was observed: 9.1% at 2.5 mg/week (*n* = 1), 16.6 ± 5.3% at 5 mg/week (*n* = 20), 21.2 ± 5.1% at 7.5 mg/week (*n* = 11), and 20.2 ± 0.3% at 10 mg/week (*n* = 2) (Table S2). Given the small and unbalanced dose strata, these comparisons are descriptive and hypothesis-generating only.

Waist circumference decreased by 10.1 ± 3.7 cm (*p* < 0.0001). Changes in body weight and BMI before and after treatment are shown in Fig. 2.

All patients experienced clinically significant weight loss (≥ 5%), with 94% (*n* = 32) reaching at least 10%, 68% (*n* = 23) reaching at least 15%, and 32% (*n* = 12) reaching at least 20%.

Following treatment, notable improvements in BMI categories were observed: 29% (*n* = 10) reached normal weight, 53% (*n* = 18) became overweight, and 18% (*n* = 6) moved into the Class I obesity category. No patients remained in the Class II or III obesity categories (Fig. 1B).

### Multivariate Analysis

In exploratory multivariable linear regression including age, sex, procedure type, baseline BMI, and maintenance tirzepatide dose (*n* = 34), maintenance dose remained independently associated with higher %TBWL (B = 1.682% points per 1 mg/week increase; standardized β = 0.490; *p* = 0.0077). Overall model fit was modest (R² = 0.325; adjusted R² = 0.205; RMSE = 5.021), with a significant overall model F-test (F(5,28) = 2.702; *p* = 0.0409) (Table S3).

### Safety

No serious adverse events or treatment discontinuations occurred. All adverse events were gastrointestinal and mostly mild to moderate. Constipation was the most common (50%, *n* = 17), followed by diarrhea (8%, *n* = 3) and nausea (3%, *n* = 1). Notably, 38% (*n* = 13) of patients experienced no side effects. Adverse events reported during the study are listed in Table S4.

## Discussion

This real-world observational study suggests that tirzepatide is associated with meaningful efficacy and good tolerability in managing recurrent weight gain after BS and EBT. Our findings show an average 18% weight loss at 24 weeks. This aligns with data from the phase 3 SURMOUNT-1 trial, which indicates the substantial efficacy of tirzepatide in non-surgical obesity [9]. This result significantly surpasses the outcomes reported for other obesity-management medications in post-bariatric populations. Previous studies with liraglutide, a GLP-1 receptor agonist, demonstrated a 5–10% weight reduction in similar groups [14]. This superior performance may result from the synergistic effects of dual GIP-GLP1 receptor activation, which may be advantageous in the altered hormonal environment following bariatric surgery. Our results show greater weight loss outcomes than those reported in recent retrospective studies on tirzepatide for post-bariatric recurrent weight gain [10–12]. Several factors may account for this difference. First, excluding patients with diabetes may have selected a population more responsive to treatment. Second, the prospective, multidisciplinary approach with rigorous patient selection contrasts with the retrospective designs and broader inclusion criteria used in other studies. Concomitant lifestyle counseling and physical activity advice, delivered as part of routine post-bariatric follow-up, may have contributed to the observed weight-loss outcomes. Available evidence indicates that lifestyle interventions alone after bariatric surgery usually achieve only modest short-term weight reduction (typically < 5% at ~ 24 weeks). Consistent with the notion that lifestyle intervention may add only modest incremental weight loss once pharmacotherapy is initiated, the SURMOUNT-3 trial showed that, after a 12-week intensive lifestyle lead-in, participants continuing lifestyle with placebo had a + 2.5% weight change by week 72, whereas those receiving tirzepatide achieved an additional − 18.4%, highlighting the dominant contribution of tirzepatide to further weight reduction [15]. In this context, the weight loss observed in our cohort (− 18.1% at 24 weeks) suggests a clinically meaningful incremental effect of tirzepatide, although causality cannot be established. Randomized controlled trials comparing tirzepatide plus a standardized lifestyle program with the same lifestyle program alone are needed to determine the relative contribution of each component.

The notable change in BMI categories within our group highlights the importance of weight loss achieved. The shift of 82% of patients from obesity to a normal weight or overweight status, with no cases of moderate or severe obesity remaining, indicates a return to healthier weight ranges similar to those achieved by successful primary bariatric procedures.

These improvements are particularly significant given that recurrent weight gain post-BS is associated with increased cardiovascular risk, diabetes recurrence, and reduced quality of life [16].

Our cohort was predominantly female (76%), consistent with female predominance in bariatric surgery populations globally. In Italy, despite a lower prevalence of obesity, women are almost three times more likely than men to seek and receive surgical treatment. Our data showed no significant difference in %TBWL between sexes (females 18.3% vs. males 17.6%, *p* = 0.71). However, the small number of male participants (*n* = 8) substantially limits statistical power to detect clinically meaningful sex differences. Post-hoc analyses of the SURMOUNT clinical trial program suggest that women may achieve greater percentage weight loss with tirzepatide than men [17]. The biological basis for these potential sex-related differences remains incompletely defined. Several, potentially overlapping, mechanisms have been proposed, including sex-specific patterns of GIP receptor expression and signaling in adipose tissue, differences in the hormonal milieu (which may be particularly relevant in post–bariatric surgery physiology), and variability in incretin sensitivity [18–19]. Beyond biological factors, sex-focused obesity literature also indicates that women demonstrate higher adherence to dietary prescriptions, physical activity programs, and follow-up visits, behaviors that could amplify observed treatment effects in real-world settings [18, 20]. Future studies specifically designed and adequately powered to formally test sex-related differences, particularly in post-bariatric surgery and endoscopy populations, are warranted.

In our cohort, response to tirzepatide appeared broadly consistent across procedure categories; however, the very small numbers in some subgroups preclude drawing firm conclusions about differential effectiveness by procedure type. Given the very small sample size, findings for ESG and AGB subgroups should be considered purely descriptive and not suitable for inferential interpretation.

A similar caution applies to the dose-related findings. In this real-world cohort, a higher maintenance tirzepatide dose at week 24 was associated with greater %TBWL across descriptive dose strata and in exploratory adjusted analyses. However, dose escalation was individualized based on tolerability, clinical response, and patient preference rather than randomized. Therefore, these results should be interpreted as exploratory and hypothesis-generating and cannot support causal inferences. Residual confounding (including confounding by indication and potential reverse causality) remains possible, and the limited sample size reduces precision and limits assessment of interactions (e.g., procedure-by-dose). Larger prospective studies, ideally with standardized titration strategies or randomized dose comparisons, are needed to confirm whether a true dose-response relationship exists in the post-bariatric setting. The optimal timing of tirzepatide initiation remains uncertain from a cost-effectiveness perspective. Conceptually, earlier treatment at the onset of weight regain may help prevent progression to clinically significant relapse and the re-emergence of obesity-related complications, whereas delayed intervention is likely associated with more comorbidities, longer pharmacologic treatment, and higher total costs.

Regarding tolerability, the safety profile observed in our post-bariatric cohort is reassuring. Although theoretical concerns exist about using agents that delay gastric emptying in patients with altered gastrointestinal anatomy, especially following restrictive procedures, our experience did not reveal clinically significant adverse effects related to these mechanisms.

The gastrointestinal adverse events observed (constipation 50%, diarrhea 8%, nausea 3%) closely align with the known safety profile in non-surgical populations from the SURMOUNT trials [21], indicating that the altered gastrointestinal anatomy after BS and EBT does not significantly change the drug’s side effect profile.

Notably, 38% of patients experienced no adverse events during the 24-week treatment, a proportion higher than typically seen with other anti-obesity agents [22]. For instance, studies with orlistat report gastrointestinal side effects in up to 80% of patients, while liraglutide studies show nausea rates of up to 40%. The relatively low incidence of nausea (3%) in our group is especially significant, as this is often the most common side effect that leads to treatment discontinuation with incretin-based therapies.

Tirzepatide combines dual GIP and GLP-1 receptor agonism, which appears to reduce nausea by GIP-mediated regulation of neural pathways that counteract the nausea-inducing effects of GLP-1, enhancing treatment adherence [23]. These findings support the hypothesis that dual agonism may optimize both efficacy and tolerability in obesity pharmacotherapy.

Constipation, observed in 50% of patients, requires particular attention in the post-bariatric setting. Although it is the most common adverse event, it was generally manageable with standard interventions. Constipation did not result in any treatment discontinuations, indicating that proper patient counseling and proactive management can effectively address it.

These results have important implications for managing post-bariatric recurrent weight gain, which remains a clinical challenge [24]. The complex, relapsing nature of obesity requires multimodal strategies. Current options include intensive lifestyle and psychological interventions or revisional surgery, each with its own limitations. Lifestyle interventions alone often fail to produce sufficient weight loss, while revisional surgery involves increased surgical risk and higher costs compared to primary procedures. Compared to liraglutide, a GLP-1 receptor agonist, tirzepatide shows additional promise by activating both GIP and GLP-1 receptors, leading to improved weight-loss outcomes. The enhanced effectiveness in the post-bariatric setting may relate to the widespread presence of GIP receptors in adipose tissue, which improves metabolic flexibility and fat oxidation.

The integration of tirzepatide into post-bariatric care pathways should consider patient selection criteria and optimal timing of initiation. Early intervention at the initial signs of recurrent weight gain may improve outcomes compared with delayed treatment.

Several limitations should be acknowledged: the absence of a comparator arm is the main methodological limitation. The relatively small sample size, drawn from a single tertiary referral center, potentially limits generalizability and introduces selection bias. Additionally, the 24-week follow-up period, while sufficient to demonstrate short-term effectiveness, is insufficient to evaluate long-term outcomes. Future randomized controlled trials with larger populations and longer follow-up are necessary to validate our findings and explore impacts on obesity-related medical problems and quality of life.

Nevertheless, our study offers novel real-world insights into the potential of tirzepatide as a therapeutic option for addressing recurrent weight gain after bariatric surgery and endoscopic bariatric therapy.

## Conclusions

This study provides preliminary real-world evidence that tirzepatide may represent an effective and well-tolerated option for managing recurrent weight gain after bariatric surgery and endoscopic bariatric therapy. The observed weight loss exceeds that reported with other pharmacological interventions in this challenging population, and the favorable safety profile supports further evaluation of tirzepatide as part of multimodal management strategies for post-bariatric weight recurrence.

## Supplementary Information

Below is the link to the electronic supplementary material.

Supplementary Material 1

## Data Availability

The data presented in this study are available on request from the corresponding author.

## References

[1] Arterburn DE, Telem DA, Kushner RF, Courcoulas AP. Benefits and risks of bariatric surgery in adults: a review. JAMA. 2020;324(9):879–87. 32870301 10.1001/jama.2020.12567

[2] Syn NL, Cummings DE, Wang LZ, Lin DJ, Zhao JJ, Loh M, et al. Association of metabolic-bariatric surgery with long-term survival in adults with and without diabetes: a one-stage meta-analysis of matched cohort and prospective controlled studies with 174772 participants. Lancet. 2021;397(10287):1830–41. 33965067 10.1016/S0140-6736(21)00591-2

[3] Wiggins T, Guidozzi N, Welbourn R, Ahmed AR, Markar SR. Association of bariatric surgery with all-cause mortality and incidence of obesity-related disease at a population level: a systematic review and meta-analysis. PLoS Med. 2020;17(7):e1003206. 32722673 10.1371/journal.pmed.1003206 PMC7386646

[4] King WC, Hinerman AS, Belle SH, Wahed AS, Courcoulas AP. Comparison of the performance of common measures of weight regain after bariatric surgery for association with clinical outcomes. JAMA. 2018;320(15):1560–9. 30326125 10.1001/jama.2018.14433 PMC6233795

[5] Cooper TC, Simmons EB, Webb K, Burns JL, Kushner RF. Trends in weight regain following Roux-en-Y gastric bypass (RYGB) bariatric surgery. Obes Surg. 2015;25(8):1474–81. 25595383 10.1007/s11695-014-1560-z

[6] American Society for Metabolic and Bariatric Surgery (ASMBS). ASMBS position statement on weight regain after bariatric surgery. Surg Obes Relat Dis. 2022;18(4):375–81. 10.1016/j.soard.2021.10.023 34896011

[7] Lucas E, Simmons O, Tchang B, Aronne L. Pharmacologic management of weight regain following bariatric surgery. Front Endocrinol (Lausanne). 2023;13:1043595. 10.3389/fendo.2022.1043595. 36699042 10.3389/fendo.2022.1043595 PMC9868802

[8] Vinciguerra F, Longhitano S, Carrubba N, Piazza L, Di Stefano C, Arpi ML, Baratta R, Hagnäs M, Frittitta L. Efficacy, feasibility and tolerability of ketogenic diet for the treatment of poor response to bariatric surgery. J Endocrinol Invest. 2023;46(9):1807–14. 10.1007/s40618-023-02034-2. 36809658 10.1007/s40618-023-02034-2 PMC10371952

[9] Jastreboff AM, Aronne LJ, Ahmad NN, et al. Tirzepatide once weekly for the treatment of obesity. N Engl J Med. 2022;387(3):205–16. 35658024 10.1056/NEJMoa2206038

[10] Stoll F, Kantowski T, Laaser J, Kloiber U, Plitzko G, Mann O, Aberle J, Lautenbach A. Tackling suboptimal clinical response after metabolic bariatric surgery: impact of tirzepatide on weight loss and body composition. Obes Res Clin Pract. 2025;19(1):63–9. 39952885 10.1016/j.orcp.2025.02.004

[11] Jamal M, et al. Semaglutide and tirzepatide for the management of weight recurrence after sleeve gastrectomy: a real-world retrospective study. Obes Surg. 2024;34(4):765–73. 10.1007/s11695-024-07137-0 38430320

[12] Bahdi F, Shah S, Dahoud F, Farooq M, Kozan P, Kim S, Sedarat A, Shen N, Thaker A, Kolb JM, Dutson E, Muthusamy VR, Issa D. Revisional endoscopic sleeve gastroplasty versus semaglutide and tirzepatide for weight recidivism after sleeve gastrectomy. Clin Obes. 2025;15(3):e70001. 10.1111/cob.70001. 39909715 10.1111/cob.70001

[13] World Health Organization. Obesity and overweight. Fact sheet. Geneva: WHO; 2021.

[14] Vinciguerra F, Di Stefano C, Baratta R, Pulvirenti A, Mastrandrea G, Piazza L, Guccione F, Navarra G, Frittitta L. Efficacy of high-dose liraglutide 3.0 mg in patients with poor response to bariatric surgery: real-world experience and updated meta-analysis. Obes Surg. 2024;34(2):303–9. 10.1007/s11695-023-07053-9. 38183597 10.1007/s11695-023-07053-9 PMC10811090

[15] Wadden TA, Chao AM, Machineni S, Kushner RF, Ard JD, Srivastava G, et al. Tirzepatide after intensive lifestyle intervention in adults with overweight or obesity: the SURMOUNT-3 phase 3 trial. Nat Med. 2023;29(11):2909–18. 10.1038/s41591-023-02597-w. 37840095 10.1038/s41591-023-02597-w PMC10667099

[16] El Asari S, Mazzini GS, Dutranoy J, Husson N, Gagniere J. Definition and management of weight regain after bariatric surgery: an international consensus. Obes Surg. 2021;31(8):3493–9.

[17] García-Pérez LE, Chao AM, Taylor R, et al. Body weight reduction with tirzepatide by sex: a subgroup analysis of the SURMOUNT clinical trials. Diabetologia. 2024;67(Suppl 1):S361–2. Abstract 756.

[18] Ghanta A, Wilson E, Chao AM. Sex differences in obesity and its treatment. Curr Psychiatry Rep. 2025;27(5):278–85. 10.1007/s11920-025-01601-z. 40100584 10.1007/s11920-025-01601-z

[19] Xie C, Huang W, Sun Y, Xiang C, Trahair L, Jones KL, et al. Disparities in the glycemic and incretin responses to intraduodenal glucose infusion between healthy young men and women. J Clin Endocrinol Metab. 2023;108(9):e712–9. 10.1210/clinem/dgad176. 36987568 10.1210/clinem/dgad176 PMC10438868

[20] Setarehdan SA, Sheidaei A, Mokhber S, Varse F, Pazouki A, Solaymani-Dodaran M. Determinants of patient’s adherence to the predefined follow-up visits after bariatric surgery. Obes Surg. 2023;33(2):577–84. 10.1007/s11695-022-06428-8. 36572837 10.1007/s11695-022-06428-8 PMC9792310

[21] Aronne LJ, Sattar N, Horn DB, Bays HE, Wharton S, Lin WY, Ahmad NN, Zhang S, Liao R, Bunck MC, Jouravskaya I, Murphy MA. SURMOUNT-4 Investigators. Continued treatment with tirzepatide for maintenance of weight reduction in adults with obesity: the SURMOUNT-4 randomized clinical trial. JAMA. 2024;331(1):38–48. 10.1001/jama.2023.24945. 38078870 10.1001/jama.2023.24945 PMC10714284

[22] Vinciguerra F, Romeo LM, Frittitta L, Baratta R. Pharmacological treatment of non-responders following bariatric surgery. Minerva Endocrinol (Torino). 2024;49(2):196–204. 10.23736/S2724-6507.21.03311-3. 33792233 10.23736/S2724-6507.21.03311-3

[23] Boer GA, Hay DL, Tups A. Obesity pharmacotherapy: incretin action in the central nervous system. Trends Pharmacol Sci. 2023;44(1):50–63. 10.1016/j.tips.2022.11.001. 36462999 10.1016/j.tips.2022.11.001

[24] Noria SF, Shelby RD, Atkins KD, Nguyen NT, Gadde KM. Weight regain after bariatric surgery: scope of the problem, causes, prevention, and treatment. Curr Diab Rep. 2023;23(3):31–42. 10.1007/s11892-023-01498-z. 36752995 10.1007/s11892-023-01498-z PMC9906605

