# Supplementary material for: Tirzepatide for Recurrent Weight Gain after Bariatric Procedures: Real-World Evidence of Efficacy and Safety
Source: Obes Surg. 2026 Jun 5;36(7):3697–704. doi: 10.1007/s11695-026-08754-7 (PMC13323781; doi:10.1007/s11695-026-08754-7)
Supplement: Supplementary file 1 — Supplementary Material 1 [file 11695_2026_8754_MOESM1_ESM.docx]

**SUPPLEMENTARY TABLES**

**Table S1: Population’s baseline characteristics**

| Gender (F/M) | 26/8 |
| --- | --- |
| Age (years old) | 49.1 ± 11.1 |
| Bariatric procedure | OAGB, n=18 (53%) |
|  | SG, n=13 (38%) |
|  | AGB, n=1 (3%) |
|  | ESG n=2 (6%) |
| BMI pre bariatric procedure (kg/m2) | 42.6± 5.1 |
| BMI nadir post bariatric procedure (kg/m2) | 27.5± 3.3 |
| Time since bariatric procedure | 6 ± 3 years |

**Table S2: Total body weight loss (%TBWL) at week 24 stratified by bariatric/endoscopic procedure and by maintenance tirzepatide dose achieved at week 24.**

| **Stratification category** | **n** | **%TBWL at week 24 (mean ± SD)** |
| --- | --- | --- |
| **Procedure type** |  |  |
| OAGB | 18 | 19.0 ± 5.5 |
| SG | 13 | 18.5 ± 5.4 |
| ESG | 2 | 11.5 ± 2.0 |
| AGB | 1 | 9.1 (single observation) |
| **Maintenance tirzepatide dose (mg/week)** |  |  |
| 2.5 | 1 | 9.1 (single observation) |
| 5.0 | 20 | 16.6 ± 5.3 |
| 7.5 | 11 | 21.2 ± 5.1 |
| 10.0 | 2 | 20.2 ± 0.3 |

Values are mean ± SD unless otherwise specified. Due to very small and unbalanced subgroup sizes, comparisons across strata are descriptive.

**Table S3. Exploratory multivariable linear regression for %TBWL at week 24**

Model summary: n=34; R²=0.325; adjusted R²=0.205; RMSE=5.021; F(5,28)=2.702; p=0.0409

| Predictor | B (unstandardized) | SE | 95% CI | Standardized β | T | p |
| --- | --- | --- | --- | --- | --- | --- |
| Intercept | 3.236 | 10.682 | −18.65 to 25.12 | - | 0.303 | 0.7642 |
| Sex (binary) | 3.628 | 2.094 | −0.66 to 7.92 | 0.277 | 1.732 | 0.0942 |
| Age (years) | −0.098 | 0.079 | −0.26 to 0.06 | −0.194 | −1.235 | 0.2270 |
| Procedure type (covariate) | −0.711 | 1.887 | −4.58 to 3.15 | −0.064 | −0.377 | 0.7090 |
| Baseline BMI (kg/m²) | 0.214 | 0.287 | −0.37 to 0.80 | 0.121 | 0.746 | 0.4616 |
| Maintenance dose at week 24 (mg/week) | 1.682 | 0.586 | 0.48 to 2.88 | 0.490 | 2.869 | 0.0077 |

Exploratory/hypothesis-generating analysis. Maintenance dose reflects individualized, non-randomized titration in routine care; therefore, the observed association should not be interpreted causally. Categorical predictors were coded as in the analysis dataset

**Table S4: Adverse events reported during the study**

| Constipation | 50% (n=17) |
| --- | --- |
| Diarrhea | 8% (n=3) |
| Nausea | 3% (n=1) |
